# Quantitative Analysis of the Multicomponent and Spectrum–Effect Correlation of the Antispasmodic Activity of Shaoyao-Gancao Decoction

**DOI:** 10.1155/2022/2279404

**Published:** 2022-12-02

**Authors:** Yanli Xu, Chenxi Li, Ting Chen, Xiaochun Li, Xiaoyu Wu, Qili Zhang, Lei Zhao

**Affiliations:** ^1^Gansu University of Chinese Medicine, Lanzhou 730000, China; ^2^Lanzhou Institute for Food and Drug Control, Lanzhou 730000, China; ^3^Northwest Collaborative Innovation Center for Traditional Chinese Medicine Co-Constructed By Gansu Province & MOE of PRC, Lanzhou 730000, China; ^4^Key Laboratory of Chemistry and Quality of TCM of the College of Gansu Province, Lanzhou 730000, China; ^5^Gansu Province Engineering Laboratory for TCM Standardization Technology and Popularization, Lanzhou 730000, China

## Abstract

Shaoyao-Gancao Decoction (SGD) is a well-known classic traditional Chinese medicine (TCM) with antispasmodic, anti-inflammatory, and analgesic effects. This preparation has been widely used to treat spasticity diseases in the clinic. To date, the material basis of SGD remains unclear, and the spectrum-effect correlation of its antispasmodic activity has not been reported yet. In this study, high-performance liquid chromatography (HPLC) was used to establish the fingerprint and determine the multiple components of SGD. The common peaks of fingerprints were evaluated by the similarity with the chromatographic fingerprints of the TCM. Meanwhile, the multiple components were quantified and analysed using the heatmap and box size analysis. Furthermore, data on the antispasmodic effect were extracted through in vitro smooth muscle contraction assay. Grey relational analysis combined with partial least square regression was used to study the spectrum–effect correlation of SGD. Finally, the potential antispasmolytic components were validated using an isolated tissue experiment. The HPLC fingerprint was established, and 20 common peaks were identified. The similarities of 15 batches of SGD were all above 0.965. The HPLC method for simultaneous determination of the multiple components was accurate and reliable. The contents of albiflorin, paeoniflorin, liquiritin, and glycyrrhizic acid were higher than the other components in SGD. The heatmap and box size also showed that *X*3 (albiflorin), *X*4 (paeoniflorin), *X*5 (liquiritin), *X*11 (liquirtigenin), and *X*16 (glycyrrhizic acid) could be used as quality indicators in the further establishment of quality standards. The spectrum–effect correlation results indicated that *X*4, *X*11, and *X*16 were highly correlated with antispasmolytic activity. Verification tests showed that paeoniflorin (11.7–29.25 *μ*g/mL) and liquirtigenin (17.19–28.65 *μ*g/mL) could significantly reduce the maximum contractile (*P* < 0.01). These compounds exerted concentration-dependent spasmolytic effects with the inhibitory response for acetylcholine (Ach)-evoked contraction. Thus, SGD had a significant antispasmodic effect, which resulted from the synergistic activity of its multiple components. These findings can be used for the pharmacodynamics study of SGD and are of great significance for the determination of quality markers and quality control.

## 1. Introduction

Spasticity is a velocity-dependent increase in muscle tone caused by the increased excitability of muscle spindles following an upper motor neuron (UMN) syndrome [1, 2]. There has been much debate about the definition of spasticity. In 1980, Lance was the first scholar to associate spasticity to the velocity-dependent increase in stretch reflex [3]. A more general definition of spasticity is disordered sensory-motor control, resulting from UMN lesion, presenting intermittent or sustained involuntary activation of muscles [4]. This complex phenomenon of extremely variable clinical expression, which may cause different motor dysfunctions, has been observed in many patients with spinal cord injury, cerebral palsy, multiple sclerosis, and acquired brain injury, which directly impact the quality of life [5]. Presently, the conventional drugs used in the treatment of spasm include baclofen, tizanidine, and dantrolene, amongst others. However, the efficacy of these current treatments is not absolute, and they may have serious side effects [6–8].

Accordingly, natural products with therapeutic activity against spasm must be searched to replace drugs with strong side effects. Shaoyao-Gancao Decoction (SGD) is a classic traditional Chinese medicine (TCM) and originally described in the Treatise on Febrile Disease. SGD is composed of Paeoniae Radix Alba (baoshao in Chinese) and honeyed *Glycyrrhiza uralensis*, which are traditionally used to treat spastic diseases, such as gastrointestinal spasm, facial muscle spasm, and poststroke spasm [9]. Modern pharmacological and clinical studies have confirmed that SGD has significant antispasmodic, anti-inflammatory, and analgesic effects on various spastic diseases [10], inflammatory diseases [11], painful diseases [12, 13], gynopathy [14], bronchial asthma, Parkinson's disease, and constipation [15, 16]. This TCM has been selected for the first batch of the Chinese Medicine Classical Directory. Studies have shown that the extract of SGD and liquorice exerts a relaxant effect on acetylcholine (ACh)-induced contraction, isoliquiritigenin, and glycycoumarin isolated from the roots of liquorice and has a potent antispasmodic [17, 18]. The ethanol extract of *Glycyrrhiza uralensis* has significant inhibitory effects on Nav1.4 VGSCs, which may be an important mechanism in the treatment of gastrocnemius spasm [19].

TCM is characterised by multiple components, targets, and approaches, and SGD has a complex composition. Previous studies have shown that glycyrrhizin, glycyrrhetic acid, paeoniflorin, albiflorin, oxypaeoniflorin, liquiritin, liquiritigenin, isoliquiritin, isoliquiritigenin, and 1,2,3,4,6-*O*-pentagalloylglucose are the main bioactive compounds of SGD [20–22]. Research on SGD has mainly focused on its clinical application, chemical composition, and anti-inflammatory and analgesic effects. However, the pharmacodynamic basis of the antispasmodic effect and the spectrum–effect relationship of SGD has not been reported yet. The manner by which the components contribute to the antispasmolytic activity of SGD remains ambiguous. The spectrum–effect relationship of TCM mainly apply correlational analysis, grey correlational analysis (GRA), multiple regression analysis, partial least squares regression (PLSR), principal component analysis, and other mathematical models to screen the bioactive compounds [23–25]. It is a biological effect-based evaluation method, which has been widely used to investigate the material basis of the pharmacological effects of Chinese medicinal compounds [26–28].

Therefore, this study was conducted to clarify the material basis of the antispasmodic effect of SGD by establishing the spectrum–effect relationship, determine the multiple components of SGD, and validate the spectrum–effect results. Scientific basis for the secondary development and quality control of SGD was also provided.

## 2. Materials and Methods

### 2.1. Samples, Reagents, and Animals

Fifteen batches of SGD were purchased from drug manufacturers in Gansu Province. The sources of herbal materials used in SGD are shown in Table 1. The raw materials were identified by Renyuan Zhu (a senior engineer in the Lanzhou Institute for Food and Drug Control). Paeoniae Radix Alba is the dried root slice of *Paeonia lactiflora* Pall. Roasted liquorice is the root and rhizome of *G. uralensis* Fisch., *Glycyrrhiza inflata* Bat., or *Glycyrrhiza glabra* L. The quality of the herbal materials complied with the standards of the National 2020 Pharmacopeia.

Methanol and acetonitrile (HPLC grade) were purchased from Honeywell China Co., Ltd. (Shanghai, China). Water was ultrapure. Glycyrrhizic acid (No. 110731–202122, purity >99.06%) and catechin (No. 110877–202005, purity >95.1%) reference substances were purchased from the China Institute of Food and Drug Control (Beijing, China). Isoliquiritigenin (No. 20112401, purity >99.04%), 1,2,3,4,6-*O*-pentagalloylglucose (No. 19010904, purity >98%), ononin (No. 19071501, purity >98%), liquiritin apioside (No. 20111607, purity >98.28%), licochalcone B (No. 20121501, purity >93%), galloylpaeoniflorin (No. 171013, purity >98%), licochalcone A (No. 19102405, purity >98.89%), oxypaeoniflorin (No. 19120604, purity >99.67%), isoliquiritin (No. 20041301, purity >99.95%), liquiritigenin (No. 21052404, purity >99.86%), albiflorin (No. 21031706, purity >98.75%), glabridin (No. 21060801, purity >99.08%), benzoylpaeoniflorin (No. 20092303, purity >99.43%), and paeoniflorin (No. 20030901, purity >98.2%) reference substances were purchased from the Chengdu Grip Biotechnology Co., Ltd. (Chengdu, China). Other compounds were of analytical grade.

Male adult Sprague–Dawley (SD) rats, 3–6 months of age, and weighing 250–300 g, were obtained from the Animal Experiment Center of Gansu University of Traditional Chinese Medicine (Approval No. SCXK[Gan]2020–0009, Lanzhou, Gansu, China). The animals were housed under standard temperature, humidity, and light conditions.

### 2.2. Apparatus and Conditions

HPLC analysis of SGD was performed using Shimadzu LC-20A high performance liquid chromatograph coupled with DAD detectors (Shimadzu Corporation, Japan). The chromatographic conditions were as follows: column, CAPCELL PAK-C18 reversed-phase (250 mm × 4.6 mm, 5 *μ*m); mobile phase, acetonitrile (A), and 0.1% phosphoric acid in water (B); flow rate, 1.0 mL/min; detection wavelength, 254 nm; column temperature, 30°C; and injection volume, 10 *μ*L. The gradient programme is shown in Table 2.

### 2.3. Solution Preparation

#### 2.3.1. Standard Solution Preparation

Certain amounts of the 15 reference standards were accurately weighed, individually placed in a 10 mL volumetric flask, and dissolved with methanol to prepare stock solutions. A certain amount of each stock solution was placed in a 10 mL volumetric flask and diluted to volume with methanol at the following concentrations: oxypaeoniflorin, 14.01 *μ*g/mL; catechin, 33.00 *μ*g/mL; albiflorin, 93.94 *μ*g/mL; paeoniflorin, 130.2 *μ*g/mL; liquiritin, 138.1 *μ*g/mL; galloylpaeoniflorin, 12.60 *μ*g/mL; 1,2,3,4,6-*O*-pentagalloylglucose, 17.72 *μ*g/mL; ononin, 17.66 *μ*g/mL; isoliquiritin, 15.29 *μ*g/mL; licochalcone B, 1.68 *μ*g/mL; liquiritigenin, 25.11 *μ*g/mL; benzoylpaeoniflorin, 2.35 *μ*g/mL; glycyrrhizic acid, 98.19 *μ*g/mL; licochalcone A, 15.91 *μ*g/mL; and glabridin, 3.63 *μ*g/mL. The mixed standard solution was diluted stepwise with methanol solution to obtain six different concentrations for the plotting of the calibration curves. All standard solutions were stored at 4°C.

#### 2.3.2. Sample Solution Preparation

The daily dose of SGD pieces (55.2 g) was precisely weighed, and 600 mL of water was added each time. The solution was decocted to ∼300 mL for 2 h and filtered. The filtrates were combined. Then, 200 mL of decoction was freeze-dried, and the other 100 mL of decoction was concentrated to 1 g/mL as a sample for the isolated smooth muscle experiment. The SGD freeze-dried powder (0.1 g) was precisely weighed, placed in a 10 mL volumetric flask, and ultrasonically extracted with 50% methanol. The sample solution was filtered through a 0.45 *μ*m membrane and stored at 4°C.

### 2.4. Validation of HPLC Analytical Method

The blank solvent (50% methanol), standard solution, negative sample, and sample solution were separately injected according to the chromatographic conditions under Section 2.2. The chromatographic results were recorded. The calibration curves were plotted with the concentration of tested reference as the *x*-axis and the peak area as the *y*-axis. The intraday and interday precisions were determined by six repetitive injections on the same day and for three consecutive days. The stability test was evaluated by injecting the sample solution at 0, 2, 4, 6, 8, 10, and 12 h after preparation. Repeatability was determined by analysing six prepared samples from the same source. Recovery was investigated by adding an accurate amount of standard solution to 0.1 g of the freeze-dried powder. Fifteen samples were prepared in parallel according to the preparation method of the sample solution.

### 2.5. Isolated Rat Intestine Preparation

The SD rats were fasted for 24 h and drank water freely. The rats were killed following a blow on the back of the head with a wooden stick [29]. The intestine segments (1.5 cm long) were prepared, gently flushed with Tyrode buffer, and quickly placed in a Petri dish containing Tyrode buffer. According to the physiological position from top to bottom, the upper end was connected to the tension transducer, and the lower end was fixed to the L-shaped bent hook at the bottom of the muscle groove. Each intestine segment was suspended in organ baths containing constantly oxygenated Tyrode's solution (20 mL, pH 8.2) at a constant temperature (37°C ± 0.5°C) [30]. Fresh oxygen was continuously introduced at a rate of 1–2 bubbles per second. The intestine segments were equilibrated for 55 ± 5 min with drainage of the buffer with fresh oxygen after 15 ± 2 min. The physiological response of the intestine segments was recorded using an isometric force transducer (ML870) connected to a 4-channel bridge amplifier. The signals were amplified by a data acquisition device Power Lab 8/35 hardware. Muscle contractions were analysed using Lab Chart 8 software. The equipment hardware and software were from ADInstruments Pty Ltd. (Bella Vista, NSW, Australia) [31].

The possible antispasmodic activity of SGD was determined by ACh (1 mM)-evoked contraction of the intestinal smooth muscle. SGD was applied cumulatively to achieve a concentration-dependent inhibitory response, and the average tension was used as the index.

### 2.6. Statistical Analysis

The chromatographic data of the 15 SGD samples were evaluated using the Chromatographic Fingerprint Evaluation System for Chinese Medicine. Graph Pad Prism (8.0.) was applied for all statistical analyses and plotting of graphs. The experimental values were expressed as mean ± standard (SEM) and tested by one-way ANOVA. *P* < 0.05 was considered to be a significant difference. GRA and PLSR were used to analyse the spectrum–effect.

## 3. Results

### 3.1. Establishment and Similarity Analysis of the HPLC Fingerprint

**Figure 1:**
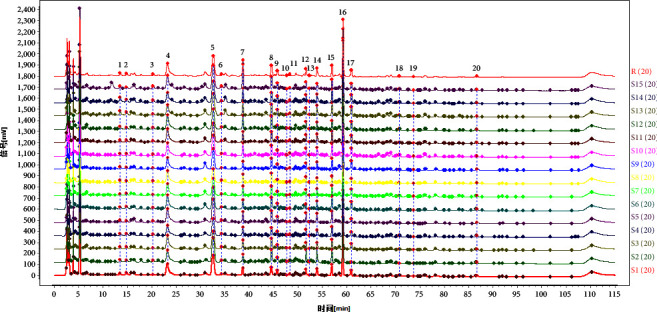
HPLC fingerprints of 15 batches of Shaoyao-Gancao Decoction (SGD; *S*1–*S*15) and control fingerprints (R).

The chromatographic data of 15 batches of SGD were imported into the Chinese Medicine Chromatographic Fingerprint Similarity Evaluation System (version 2012). After chromatographic peak matching, the standard fingerprint chromatogram “R” was generated, and the fingerprints of the 15 batches of SGD samples were established (Figure 1). The similarities between the sample chromatograms and the reference chromatogram were calculated using the abovementioned software. The similarities were all greater than 0.965 (Table 3), indicating apparent similarity amongst the 15 batches of SGD. Then, 20 common peaks in the reference chromatogram were assigned, and 16 compounds, including oxypaeoniflorin, catechin, albiflorin, paeoniflorin, liquiritin, galloypaeoniflorin, 1,2,3,4,6-*O*-pentagalloylglucose, ononin, isoliquiritin, licochalcone B, liquirtigenin, benzoylpaeoniflorin, glycyrrhizic acid, licochalcone A, glabridin, and glycyrrhetinic acid, were verified after a comparison with the reference substances.

Components: 1, oxypaeoniflorin; 2, catechin; 3, albiflorin; 4, paeoniflorin; 5, liquiritin; 6, galloypaeoniflorin; 7, 1,2,3,4,6-*O*-pentagalloylglucose; 8, ononin; 9, isoliquiritin; 10, licochalcone B; 11, liquirtigenin; 12, benzoylpaeoniflorin; 16, glycyrrhizic acid; 18, licochalcone A; 19, glabridin; and 20, glycyrrhetinic acid.

### 3.2. Validation of the HPLC Method

A method that could distinguish the HPLC fingerprint and simultaneously determine the 15 compounds was established. The validation of the method, including precision, repeatability, stability, linear regression, and recovery for 15 compounds, is summarised in Table 4. The results showed that the precision of the instrument and the repeatability of the extraction method were good, and the sample was stable within 12 h. All calibration curves showed good linearity in the given concentration ranges. The recovery rates for the spiked samples ranged from 93.9% to 109.9%. Thus, the validation of the HPLC method was within an acceptable range in quantitative research, demonstrating that the established method was reproducible for the fingerprint and the determination of 15 compounds in different batches of SGD. The proposed method can simultaneously determine 15 compounds and can provide a better alternative for the evaluation of the quality of SGD.

### 3.3. Measurement Results of Multiple Component Determination

**Figure 2:**
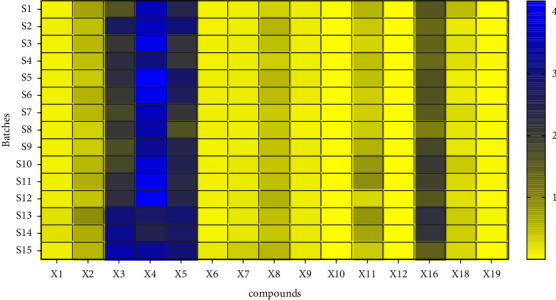
Heatmap of 15 compounds in 15 batches of SGD.

**Figure 3:**
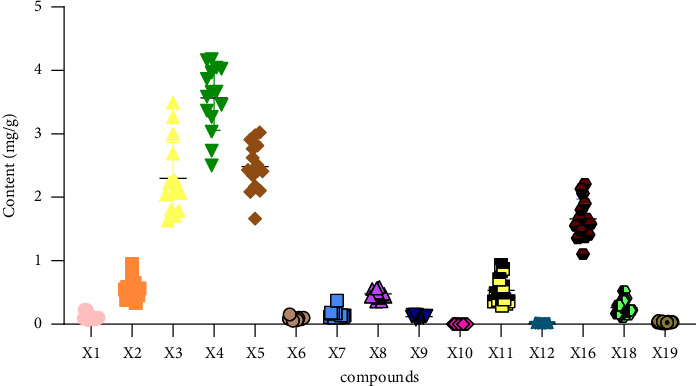
Box plot of 15 compounds in 15 batches of SGD.

**Figure 4:**
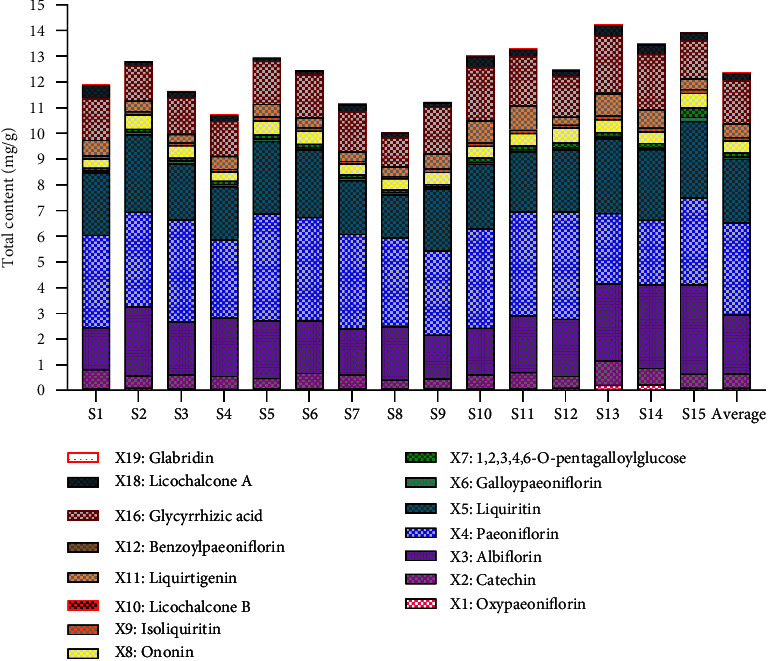
Bar graph of the total contents in 15 batches of SGD.

The quantities of the 15 components measured in the SGD were calculated by substituting the regression equation in Table 4.  Table 5 shows that the content of the 15 compounds in the different batches of SGD varied to a certain extent. The fact that the raw materials were derived from different sources may be the main reason for the fluctuation in the content of the tested compounds. We adopted a heatmap and box plot to intuitively display the content distribution [32]. The heatmap reflected the fluctuation of the 15 compounds in different batches through the gradient colour. As shown in Figure 2, *X*3 (albiflorin), *X*4 (paeoniflorin), *X*5 (liquiritin), and *X*16 (glycyrrhizic acid) fluctuated obviously, reflecting great variation amongst the different batches. The box size represents the dispersion degree of the 15 index compounds in the different batches. As shown in Figure 3, *X*2 (catechin), *X*3 (albiflorin), *X*4 (paeoniflorin), *X*5 (liquiritin), *X*11 (liquirtigenin), and *X*16 (glycyrrhizic acid) were relatively large. As required by ChP, the quality of liquorice and Paeoniae Radix Alba were evaluated by detecting the content of liquiritin, glycyrrhizic acid, and paeoniflorin. As shown in Figure 4, the total contents were markedly different, and the total contents of *S*2, *S*5, *S*6, and *S*10–*S*15 were higher than the average. Furthermore, their quality was better than the other contents. The average contents of 15 characteristic ingredients in SGD from high to low were as follows: *X*4 > *X*5 > *X*3 > *X*16 > *X*2 > *X*11 > *X*8 > *X*18 > *X*7 > *X*9 > *X*1 > *X*6 > *X*19 > *X*12 > *X*10. Thus, we suggest that *X*3 (albiflorin), *X*4 (paeoniflorin), *X*5 (liquiritin), *X*11 (liquirtigenin), and *X*16 (glycyrrhizic acid) can be used as characteristic components when a quality standard is established.

### 3.4. Results of Isolated Intestine Preparation

In the intestinal muscle study, Ach-induced intestine contractions were used to evaluate the antispasmolytic activity of the SGD samples.  Table 6 shows that compared with the blank control group, the intestine contractions of the Ach model group were significantly increased (*P* < 0.05), indicating that the model was successful. Compared with the Ach model group, 15 batches of SGD (25 mg/mL and 35 mg/mL) from different origins all significantly reduced the maximum contractile (*P* < 0.01), exerting concentration-dependent spasmolytic effects with the inhibitory response for Ach-evoked contraction.

### 3.5. Spectral-Effect Relevance Analysis

GRA is a quantitative analytical method widely used to analyse the correlation between the compound and its efficacy [16]. A correlation coefficient greater than 0.8 indicates a strong correlation [33]. The common peaks and the inhibition rate of SGD on the intestinal contraction after *Z*-score normalisation were used as the *X* and *Y* matrices, respectively, in the GRA to find the active compounds corresponding to the antispasmolytic efficacy. The results of the GRA are shown in Table 7. The correlation coefficients of *X*4 (paeoniflorin), *X*15, *X*14, *X*8 (ononin), *X*6 (galloypaeoniflorin), *X*5 (liquiritin), *X*9 (isoliquiritin), and *X*16 (glycyrrhizic acid) were higher than 0.8, indicating their major role in the antispasmolytic activity of SGD. These results also signify that SGD exerted antispasmolytic effects through multicomponent synergy.

#### 3.5.1. PLSR

**Figure 5:**
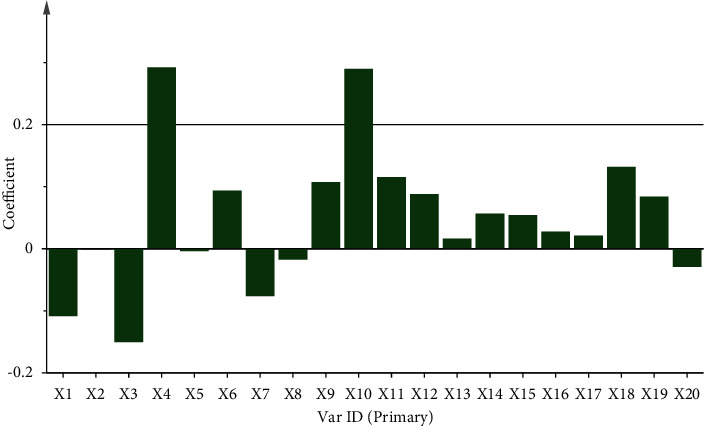
Coefficient of correlation of 20 peaks with antispasmolytic activity.

In addition to GRA, the relationship between the 20 common peaks (*x*-variables) and the antispasmolytic efficacy (*y*-variables) was also evaluated by using a PLSR model. PLSR is a method that can describe which peaks contribute positively or negatively to the efficacy. As shown in Figure 5, the peaks *X*4, *X*6 and *X*9–*X*19 were correlated strongly with the antispasmolytic effect with high positive correlation coefficients. The remaining seven peaks were negatively correlated with the inhibition rate.

**Figure 6:**
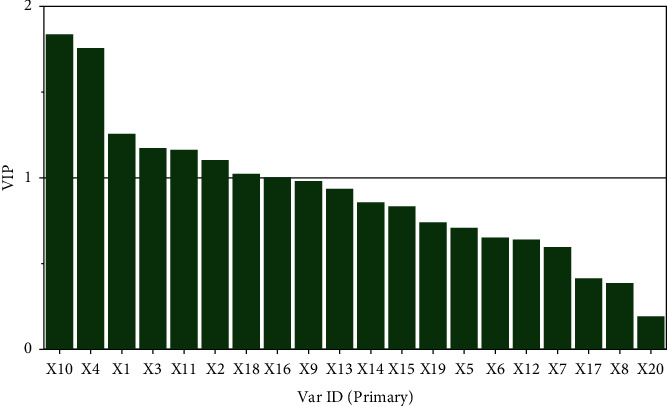
VIP contribution plot of common peak antispasmolytic activity.

Furthermore, the VIP value can describe the degree of explanation of the independent variable on the dependent variable. The larger the VIP value, the greater the correlation between the variable and the drug efficacy will be [34]. When the VIP is greater than 1, the characteristic peak has a more important role in the antispasmolytic efficacy. As shown in Figure 6, the VIP values of peaks *X*10, *X*4, *X*1, *X*11, *X*3, *X*2, *X*18, and *X*16 were greater than 1, indicating biological significance.

Combining the results from the GRA and PLSR models, correlation degree >0.8, VIP > 1 and positive correlation, the peaks of *X*4 (paeoniflorin), *X*11 (liquirtigenin), and *X*16 (glycyrrhizic acid) were identified as the major core antispasmolytic compounds in SGD. This finding also indicated that the antispasmodic effect of SGD is the result of the synergistic effect of multiple components. Many studies have shown that glycyrrhizic acid and paeoniflorin have synergistic effects on antispasticity, antipyretic, anti-inflammatory, inhibition of gastric secretion, and relaxation of smooth muscle [35].

#### 3.5.2. Experimental Verification

**Figure 7:**
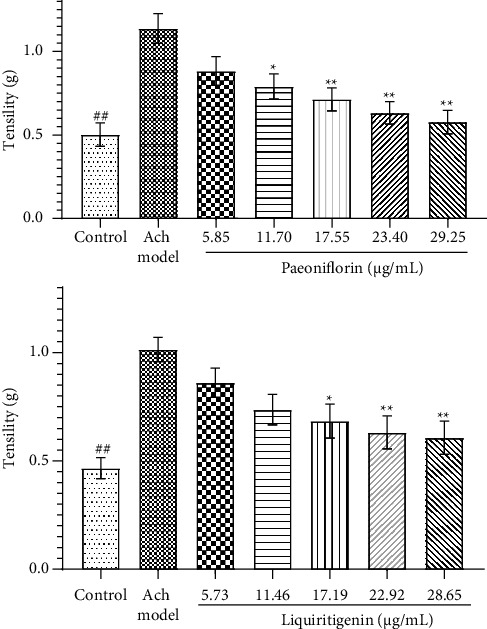
Effects of paeoniflorin and liquirtigenin on the contractile activity (*X* ± SME, *n* = 6).

Three antispasmolytic active compounds, namely, paeoniflorin, liquirtigenin, and glycyrrhizic acid, were obtained by spectral–effect relationship. Isolated intestine preparation was used to further verify the feasibility and accuracy of the spectral–effect relationship method. As shown in Figure 7, paeoniflorin (11.70–29.25 *μ*g/mL) and liquirtigenin (17.19–28.65 *μ*g/mL) could significantly reduce the maximum contractile (*P* < 0.01) and exerted concentration-dependent spasmolytic effects with the inhibitory response for Ach-induced contraction.

Compared with the control group, ^#^*P* < 0.05; compared with the Ach model ^*∗*^*P* < 0.01.

## 4. Conclusion

In this study, 15 compounds in SGD were simultaneously quantified using the HPLC analytical method. The method was simple, rapid, and accurate, and it can be used for the qualitative and quantitative analysis of SGD. The heatmap and the box sizes showed that albiflorin, paeoniflorin, liquiritin, liquirtigenin, and glycyrrhizic acid could be used as quality markers in the establishment of a quality standard. These compounds were responsible for the efficacy and treatment effect of SGD. Meanwhile, for the first time, the fingerprint of SGD was associated with antispasmolytic activity. Paeoniflorin, liquirtigenin, and glycyrrhizic acid were highly correlated with antispasmolytic activity, revealing that multiple components exhibited antispasmolytic activities. Furthermore, the potential antispasmolytic components were validated. Paeoniflorin (11.70–29.25 *μ*g/mL) and liquirtigenin (17.19–28.65 *μ*g/mL) could significantly reduce the maximum contractile (*P* < 0.01) and exerted concentration-dependent spasmolytic effects with the inhibitory response for ACh-induced contraction. Thus, SGD had a significant antispasmodic effect, which could have resulted from the synergistic activity of its paeoniflorin, liquirtigenin and other components.

The results of the quantitative analysis of the multicomponent in SGD could help to discover the material basis of SGD and establish a system for modern TCM quality standards. The spectral–effect study can be used for the rapid screening of potential antispasmolytic components in TCM. Verification experiment was carried out by testing the activity of the single standards to further determine the antispasmodic efficacy of SGD. The results can provide a reference for the pharmacodynamics study of SGD and are highly significant for the determination of quality markers and quality control. Given that TCM is characterised by multiple components, targets, and approaches, further study on the spasmolytic targets and mechanism of SGD is needed.

## Figures and Tables

**Table 1:** Source information of 15 batches of Shaoyao-Gancao Decoction (SGD).

No.	Decoction pieces	Origin	Batch no.
*S*1	Paeoniae Radix Alba	Anhui	2011007
	*G. Uralensis*, honeyed	Gansu	20190227

*S*2	Paeoniae Radix Alba	Anhui	210811
	*G. uralensis*, honeyed	Xinjiang	20201119

*S*3	Paeoniae Radix Alba	Anhui	21111604
	*G. uralensis*, honeyed	Gansu	20092201

*S*4	Paeoniae Radix Alba	Anhui	20201203
	*G. uralensis*, honeyed	Neimeng	200923

*S*5	Paeoniae Radix Alba	Anhui	21100701
	*G. uralensis*, honeyed	Gansu	22022301

*S*6	Paeoniae Radix Alba	Anhui	21102303
	*G. uralensis*, honeyed	Gansu	210801

*S*7	Paeoniae Radix Alba	Anhui	21031306
	*G. uralensis*, honeyed	Gansu	20211202

*S*8	Paeoniae Radix Alba	Anhui	2110087
	*G. uralensis*, honeyed	Gansu	210605

*S*9	Paeoniae Radix Alba	Anhui	2003011
	*G. uralensis*, honeyed	Gansu	2107006

*S*10	Paeoniae Radix Alba	Anhui	2010077
	*G. uralensis*, honeyed	Gansu	2011011

*S*11	Paeoniae Radix Alba	Anhui	07220051
	*G. uralensis*, honeyed	Gansu	2009020

*S*12	Paeoniae Radix Alba	Anhui	07220045
	*G. uralensis*, honeyed	Gansu	20033102

*S*13	Paeoniae Radix Alba	Zhejiang	20211025007
	*G. uralensis*, honeyed	Gansu	2104011

*S*14	Paeoniae Radix Alba	Zhejiang	20211021001
	*G. uralensis*, honeyed	Gansu	202202001

*S*15	Paeoniae Radix Alba	Anhui	21022301
	*G. uralensis*, honeyed	Gansu	202108004

**Table 2:** Gradient program.

Time (min)	*A* (%)	*B* (%)
0	90	10
30	80	20
60	50	50
85	25	75
90	10	90
100	10	90
105	90	10

**Table 3:** Similarity between the sample and control fingerprints.

No.	Similarity
*S*1	0.994
*S*2	0.968
*S*3	0.993
*S*4	0.995
*S*5	0.989
*S*6	0.989
*S*7	0.996
*S*8	0.984
*S*9	0.988
*S*10	0.993
*S*11	0.995
*S*12	0.995
*S*13	0.983
*S*14	0.982
*S*15	0.965

**Table 4:** Linear regression, precision, repeatability, stability, and recovery for 15 components in SGD.

Compound	Regression equation	Correlation coefficient (*r*)	Linear range (mg/mL)	Precision (RSD, %) *n* = 5	Repeatability (RSD, %) *n* = 6	Stability (RSD, %) *n* = 6	Recovery (RSD, %) *n* = 6
*X*1: oxypaeoniflorin	*Y* = 1233273.12*X* + 46449.80	0.9944	0.056–0.559	0.9	2.0	0.5	96.8 ± 0.4
*X*2: catechin	*Y* = 213488.20*X* + 9856.90	0.9983	0.126–1.255	2.4	0.4	2.5	96.8 ± 1.8
*X*3: albiflorin	*Y* = 31301.02*X* + 3371.21	0.9995	0.371–3.711	2.8	1.8	2.8	97.0 ± 1.5
*X*4: paeoniflorin	*Y* = 353199.09*X* + 4111.98	0.9999	0.512–5.117	3.6	0.7	2.5	99.3 ± 1.4
*X*5: liquiritin	*Y* = 443973.23 *X*−114367.63	0.9985	0.543–5.430	1.2	1.5	0.8	98.7 ± 1.2
X6: galloypaeoniflorin	*Y* = 931967.24*X* + 12146.77	0.9990	0.050–0.494	2.9	0.2	1.3	109.9 ± 1.7
*X*7: 1,2,3,4,6-O-pentagalloylglucose	*Y* = 3542609.33*X* + 9102.26	1.0000	0.069–0.695	1.0	1.4	0.4	101.8 ± 1.9
*X*8: ononin	*Y* = 937109.60*X* + 2791.93	1.0000	0.069–0.692	0.6	3.1	0.3	101.2 ± 0.5
*X*9: isoliquiritin	*Y* = 1557077.22*X* + 3899.99	0.9999	0.061–0.611	0.8	2.1	0.7	108.7 ± 0.5
*X*10: licochalcone B	*Y* = 1291329.69*X* + 338.63	0.9999	0.006–0.063	0.8	2.9	0.4	93.8 ± 0.5
*X*11: liquirtigenin	*Y* = 132434.69*X*–1198.43	0.9998	0.100–1.000	0.5	1.7	0.3	107.3 ± 0.4
*X*12: benzoylpaeoniflorin	*Y* = 1537913.99 *X* + 1164.36	1.0000	0.009–0.093	2.5	1.5	2.7	104.1 ± 1.1
*X*16: glycyrrhizic acid	*Y* = 858537.93*X* + 3267.07	0.9999	0.389–3.890	0.7	0.4	0.4	97.3 ± 0.1
*X*18: licochalcone A	*Y* = 137500.90*X*–5234.42	0.9979	0.063–0.629	0.4	2.1	0.3	105.4 ± 0.6
*X*19: glabridin	*Y* = 563254.16*X* + 431.74	0.9999	0.072–0.720	1.1	3.9	0.6	106.9 ± 0.5

**Table 5:** Content determination of the 15 compounds in 15 batches of SGD.

Compound	Content (mg/g)														
	*S*1	*S*2	*S*3	*S*4	*S*5	*S*6	*S*7	*S*8	*S*9	*S*10	*S*11	*S*12	*S*13	*S*14	*S*15
*X*1: oxypaeoniflorin	0.080	0.104	0.083	0.070	0.090	0.092	0.077	0.088	0.094	0.086	0.102	0.113	0.221	0.229	0.113
*X*2: catechin	0.753	0.490	0.546	0.501	0.410	0.591	0.563	0.335	0.381	0.552	0.625	0.457	0.950	0.652	0.544
*X*3: albiflorin	1.634	2.690	2.067	2.265	2.238	2.044	1.789	2.079	1.705	1.807	2.200	2.225	3.000	3.259	3.490
*X*4: paeoniflorin	3.586	3.666	3.966	3.035	4.161	4.028	3.659	3.456	3.266	3.866	4.042	4.175	2.737	2.500	3.365
*X*5: liquiritin	2.431	3.023	2.172	2.084	2.814	2.623	2.105	1.664	2.417	2.508	2.348	2.411	2.912	2.764	2.976
*X*6: galloypaeoniflorin	0.082	0.096	0.108	0.087	0.103	0.100	0.102	0.101	0.073	0.083	0.085	0.095	0.064	0.055	0.157
*X*7: 1,2,3,4,6-*O*-pentagalloylglucose	0.103	0.111	0.116	0.116	0.140	0.116	0.120	0.105	0.104	0.168	0.144	0.174	0.178	0.181	0.374
*X*8: ononin	0.360	0.575	0.497	0.366	0.555	0.518	0.422	0.437	0.474	0.454	0.482	0.591	0.503	0.436	0.568
*X*9: isoliquiritin	0.128	0.121	0.104	0.100	0.153	0.140	0.098	0.072	0.117	0.135	0.117	0.101	0.136	0.152	0.151
*X*10: licochalcone B	0.002	0.003	0.007	0.004	0.002	0.004	0.003	0.002	0.002	0.001	0.007	0.007	0.006	0.004	0.001
*X*11: liquirtigenin	0.588	0.401	0.323	0.497	0.488	0.362	0.365	0.382	0.597	0.843	0.936	0.294	0.871	0.711	0.392
*X*12: benzoylpaeoniflorin	0.007	0.005	0.005	0.008	0.004	0.008	0.007	0.011	0.010	0.011	0.012	0.013	0.016	0.016	0.020
*X*16: glycyrrhizic acid	1.615	1.367	1.410	1.358	1.663	1.663	1.554	1.108	1.803	2.060	1.904	1.583	2.209	2.131	1.477
*X*18: licochalcone A	0.521	0.151	0.223	0.232	0.115	0.158	0.274	0.196	0.167	0.410	0.263	0.218	0.390	0.370	0.292
*X*19: glabridin	0.026	0.014	0.028	0.039	0.014	0.016	0.037	0.029	0.028	0.049	0.040	0.033	0.051	0.042	0.026

**Table 6:** Effect of 15 batches of SGD on the contractile activity of isolated intestinal preparation (*X* ± SME, *n* = 6).

Batch	Average tension (g)					Inhibition rate (%)	
	Control group	Ach model group	Drug administration group (15 mg/mL)	Drug administration group (25 mg/mL)	Drug administration group (35 mg/mL)	Drug administration group (25 mg/mL)	Drug administration group (35 mg/mL)
*S*1	0.4483 ± 0.04920	1.426 ± 0.1244^##^	1.341 ± 0.0984	0.6487 ± 0.0472^*∗∗*^	0.4776 ± 0.0430^*∗∗*^	78.02 ± 5.549	97.05 ± 4.003
*S*2	0.5204 ± 0.01452	1.168 ± 0.1892^#^	1.194 ± 0.1848	0.6943 ± 0.1398	0.4543 ± 0.06812^*∗∗*^	82.90 ± 12.32	120.3 ± 9.962
*S*3	0.4285 ± 0.03723	1.216 ± 0.1397^##^	0.9784 ± 0.1218	0.5199 ± 0.05595^*∗∗*^	0.3260 ± 0.02184^*∗∗*^	88.08 ± 3.246	114.2 ± 2.65
*S*4	0.5635 ± 0.06599	1.914 ± 0.3118^##^	1.703 ± 0.2831	0.8390 ± 0.07993^*∗∗*^	0.5414 ± 0.05365^*∗∗*^	86.11 ± 9.202	111 ± 8.952
*S*5	0.4279 ± 0.06277	1.358 ± 0.1667^##^	1.160 ± 0.1562	0.5640 ± 0.08049^*∗∗*^	0.4190 ± 0.06737^*∗∗*^	84.74 ± 5.133	101.6 ± 2.695
*S*6	0.5296 ± 0.04119	1.159 ± 0.09607^##^	0.9905 ± 0.09806	0.6552 ± 0.05702^*∗∗*^	0.5028 ± 0.03212^*∗∗*^	82.45 ± 5.394	103.9 ± 3.426
*S*7	0.4323 ± 0.06757	1.124 ± 0.1909^##^	0.7886 ± 0.05031	0.3899 ± 0.03225^*∗∗*^	0.2655 ± 0.0438^*∗∗*^	99.13 ± 9.353	120.2 ± 7.817
*S*8	0.5116 ± 0.04533	0.9918 ± 0.05947^##^	1.001 ± 0.03622	0.7059 ± 0.02238^*∗∗*^	0.5774 ± 0.01672^*∗∗*^	58.10 ± 8.560	85.36 ± 7.416
*S*9	0.4842 ± 0.05229	1.063 ± 0.1186^#^	0.984 ± 0.2091	0.6202 ± 0.09016	0.3968 ± 0.0398^*∗∗*^	75.91 ± 7.580	113.4 ± 6.972
*S*10	0.4960 ± 0.05865	1.409 ± 0.1962^##^	0.9719 ± 0.08651^*∗*^	0.4914 ± 0.04814^*∗∗*^	0.368 ± 0.05182^*∗∗*^	104.2 ± 5.288	120.4 ± 10.35
*S*11	0.3607 ± 0.04392	0.9241 ± 0.1457^##^	0.5863 ± 0.07636	0.3203 ± 0.04721^*∗∗∗*^	0.2587 ± 0.05415^*∗∗*^	110.2 ± 4.840	117.5 ± 10.68
*S*12	0.3917 ± 0.03975	1.433 ± 0.2150^##^	1.249 ± 0.2104	0.6738 ± 0.1062^*∗∗*^	0.4462 ± 0.07728^*∗∗*^	75.15 ± 5.218	98.39 ± 5.121
*S*13	0.5547 ± 0.09259	1.113 ± 0.1566^#^	0.9223 ± 0.1112	0.5019 ± 0.0944^*∗∗*^	0.4470 ± 0.09103^*∗∗*^	107.4 ± 7.044	118 ± 6.441
*S*14	0.4113 ± 0.03592	0.8625 ± 0.08512^##^	0.6173 ± 0.05789^*∗*^	0.3951 ± 0.03806^*∗∗*^	0.3036 ± 0.02892^*∗∗*^	108.9 ± 7.633	127.4 ± 5.618
*S*15	0.4967 + 0.02877	1.243 ± 0.2305^#^	1.117 ± 0.2264	0.7182 ± 0.1251	0.5096 ± 0.06343^*∗*^	67.52 ± 9.100	94.27 ± 5.152

Compared with the control group: ^#^*P* < 0.05; ^##^*P* < 0.01; compared with the Ach model group: ^*∗*^*P* < 0.05; ^*∗∗*^*P* < 0.01.

**Table 7:** GRA results.

Peaks	Correlation	Ranking
*X*4	0.849	1
*X*15	0.843	2
*X*14	0.841	3
*X*8	0.836	4
*X*6	0.835	5
*X*5	0.834	6
*X*9	0.82	7
*X*16	0.812	8
*X*12	0.794	9
*X*7	0.784	10
*X*1	0.776	11
*X*3	0.774	12
*X*2	0.772	13
*X*17	0.77	14
*X*18	0.734	15
*X*19	0.728	16
*X*11	0.685	17
*X*20	0.668	18
*X*13	0.656	19
*X*10	0.653	20

## Data Availability

The data used to support the findings of this study are included within the article.

## References

[1] Lynn B.-O., Guy M., Erwin A. (2015). Spasticity and its contribution to hypertonia in cerebral palsy. *BioMed Research International*.

[2] Yang E., Lew H.-L., Özçakar L., Wu C.-H. (2021). Recent advances in the treatment of spasticity: extracorporeal shock wave therapy. *Journal of Clinical Medicine*.

[3] Barnes M.-P., Kent R.-M., Semlyen J.-K., McMullen K.-M. (2003). Spasticity in multiple sclerosis. *Neurorehabilitation and Neural Repair*.

[4] Pandyan A.-D., Gregoric M., Barnes M. P. (2005). Spasticity: clinical perceptions, neurological realities and meaningful measurement. *Disability & Rehabilitation*.

[5] Vinolo-Gil M.-J., Rodríguez-Huguet M., García-Muñoz C., Gonzalez-Medina G., Martin-Vega F. J., Martin-Valero R. (2022). Effects of peripheral electromagnetic fields on spasticity: a systematic review. *Journal of Clinical Medicine*.

[6] Pedersen E., Arlien-Soborg P., Mai J. (2009). The mode of action of the Gaba derivative baclofen in human spasticy. *Acta Neurologica Scandinavica*.

[7] Gelber D.-A., Good D. C., Dromerick A., Sergay S., Richardson M. (2001). Open-label dose-titration safety and efficacy study of tizanidine hydrochloride in the treatment of spasticity associated with chronic stroke. *Stroke*.

[8] Ketel W.-B., Kolb M. E. (1984). Long-term treatment with dantrolene sodium of stroke patients with spasticity limiting the return of function. *Current Medical Research and Opinion*.

[9] Qu Y.-Z., Ma S.-J., Zhu G.-W. (2020). Historical evolution and modern research on shaoyao gancaotang. *Chinese Journal of Experimental Traditional Medical Formulae*.

[10] Bi X.-L., Gong M.-R., Di L.-Q. (2014). Review on prescription compatibility of shaoyao gancao decoction and reflection on pharmacokinetic compatibility mechanism of traditional Chinese medicine prescription based on in vivo drug interaction of main efficacious components. *Evidence-based Complementary and Alternative Medicine*.

[11] Zhang Y., Jia X.-L., Yang J. (2016). Effects of shaoyao-gancao decoction on infarcted cerebral cortical neurons: suppression of the inflammatory response following cerebral ischemia-reperfusion in a rat model. *BioMed Research International*.

[12] Zheng W.-Q., Song L.-H., Li H.-J. (2008). Effect of pge-2/cAMP signaling pathway on analgesic effect of Shaoyao Gancao Decoction. *Pharmacol Clin Chin Mater Med*.

[13] Sui F., Zhou H.-Y., Meng J. (2016). A Chinese herbal decoction, shaoyao-gancao tang, exerts analgesic effect by down-regulating the TRPV1 channel in a rat model of arthritic pain. *The American Journal of Chinese Medicine*.

[14] Shao Y.-Y., Chang Z.-P., Cheng Y. (2019). Shaoyao-Gancao Decoction alleviated hyperandrogenism in a letrozole-induced rat model of polycystic ovary syndrome by inhibition of NF-*κ*B activation. *Bioscience Reports*.

[15] Wu L.-F., Li Y.-T., Tang Y.-Z. (2021). Research progress on chemical constituents and pharmacological activities of Shaoyao Gancao Decoction. *Drug Evaluation Research*.

[16] Huang R.-C., Zhao B.-B., Kong J. (2019). Effect of ShaoyaoGancao Decoction on neurotransmitter and myotonia in rat brain with Parkinson’s disease. *Acta Chinese Medicine*.

[17] Sato Y., He J. X., Nagai H., Tani T., Akao T. (2007). Isoliquiritigenin, one of the antispasmodic principles of Glycyrrhiza ularensis roots, acts in the lower part of intestine. *Biological & Pharmaceutical Bulletin*.

[18] Shi Y., Wu D., Sun Z. (2012). Analgesic and uterine relaxant effects of isoliquiritigenin, a flavone from Glycyrrhiza glabra. *Phytotherapy Research*.

[19] Zhu G., Ma S., Li X. (2018). The effect of ethanol extract of Glycyrrhiza uralensis on the voltage-gated sodium channel subtype 1.4. *Journal of Pharmacological Sciences (Tokyo, Japan)*.

[20] Tang N.-Y., Liu C.-H., Hsieh C.-T., Hsieh C. L. (2010). The anti-inflammatory effect of paeoniflorin on cerebral infarction induced by ischemia-reperfusion injury in sprague-dawley rats. *The American Journal of Chinese Medicine*.

[21] Lin Z.-J., Qiu S.-X., Wufuer A., Shum L. (2005). Simultaneous determination of glycyrrhizin, a marker component in radix Glycyrrhizae, and its major metabolite glycyrrhetic acid in human plasma by LC-MS/MS. *Journal of Chromatography B*.

[22] Goto E., He J.-X., Akao T., Tani T. (2010). Bioavailability of glycyrrhizin from Shaoyao-Gancao Tang in laxative treated rats. *Journal of Pharmacy and Pharmacology*.

[23] Qiao X., Qu C., Luo Q. (2021). UHPLC-qMS spectrum-effect relationships for Rhizoma Paridis extracts. *Journal of Pharmaceutical and Biomedical Analysis*.

[24] Tang J., Zhang Q., Wu D. (2022). Potential pharmacodynamic substances of Laportea bulbifera in treatment of rheumatoid arthritis based on serum pharmacochemistry and pharmacology. *China Journal of Chinese Materia Medica*.

[25] Chen Y., Zou S., Xu W., Sun Q., Yun L. (2020). Spectrum-effect relationship of antioxidant and anti-inflammatory activities of Laportea bulbifera based on multivariate statistical analysis. *Biomedical Chromatography*.

[26] qiu Q., Jiang L.-J., Huang C.-Y. (2022). Study on the spectrum-effect correlation of anti-inflammatory active extract of Sauropus spatulifolius beille. *Journal of Analytical Methods in Chemistry*.

[27] Wang J.-M., Peng L.-N., Shi M.-J., Li C., Zhang Y., Kang W. (2017). Spectrum effect relationship and component knockout in angelica dahurica radix by high performance liquid chromatography-Q exactive hybrid quadrupole-orbitrap mass spectrometer. *Molecules*.

[28] Liu X., Wang X., Zhu T. (2018). Study on spectrum-effect correlation for screening the effective components in Fangji Huangqi Tang basing on ultra-high performance liquid chromatography-mass spectrometry. *Phytomedicine*.

[29] Wahid M., Saqib F., Qamar M., Ziora Z.-M. (2022). Antispasmodic activity of the ethanol extract of *Citrullus lanatus* seeds: justifying ethnomedicinal use in Pakistan to treat asthma anddiarrhea. *Journal of Ethnopharmacology*.

[30] Vanegas Andrade C., Matera S., Bayley M. (2022). Antispasmodic, antidepressant and anxiolytic effects of extracts from Schinus lentiscifolius Marchand leaves. *Journal of Traditional and Complementary Medicine*.

[31] Gavilánez Buñay T.-C., Colareda G.-A., Ragone M.-I. (2018). Intestinal, urinary and uterine antispasmodic effects of isoespintanol, metabolite from Oxandra xylopioides leaves. *Phytomedicine*.

[32] Xue M.-J., Zhao Y.-T., Cui Y., Yang J., Wang Y., Chai X. (2022). Quantitative analysis of multicomponents in qufeng zhitong capsule and application of network pharmacology to explore the anti-inflammatory activity of focused compounds. *Journal of Analytical Methods in Chemistry*.

[33] Chen X.-Y., Gou S.-H., Shi Z.-Q., Xue Z. Y., Feng S. L. (2019). Spectrum-effect relationship between HPLC fingerprints and bioactive components of Radix Hedysari on increasing the peak bone mass of rat. *Journal of Pharmaceutical Analysis*.

[34] Gao S., Chen H., Zhou X. (2019). Study on the spectrum-effect relationship of the xanthine oxidase inhibitory activity of Ligustrum lucidum. *Journal of Separation Science*.

[35] Zhu G.-W., Zhang G.-J., Wang M. (2015). Research situation about origin, active components alignment and phramacological actions of Shaoyao Gancao Decoction. *China Journal of Traditional Chinese Medicine and pharmacy*.

